# Dietary Exercise as a Novel Strategy for the Prevention and Treatment of Metabolic Syndrome: Effects on Skeletal Muscle Function

**DOI:** 10.1155/2011/676208

**Published:** 2011-06-06

**Authors:** Wataru Aoi, Yuji Naito, Toshikazu Yoshikawa

**Affiliations:** ^1^Laboratory of Health Science, Graduate School of Life and Environmental Sciences, Kyoto Prefectural University, Kyoto 606-8522, Japan; ^2^Department of Gastroenterology and Hepatology, Graduate School of Medical Science, Kyoto Prefectural University of Medicine, Kyoto 602-8566, Japan

## Abstract

A sedentary lifestyle can cause metabolic syndrome to develop. Metabolic syndrome is associated with metabolic function in the skeletal muscle, a major consumer of nutrients. Dietary exercise, along with an adequate diet, is reported to be one of the major preventive therapies for metabolic syndrome; exercise improves the metabolic capacity of muscles and prevents the loss of muscle mass. Epidemiological studies have shown that physical activity reduces the risk of various common diseases such as cardiovascular disease, diabetes, and cancer; it also helps in reducing visceral adipose tissue. In addition, laboratory studies have demonstrated the mechanisms underlying the benefits of single-bout and regular exercise. Exercise regulates the expression/activity of proteins associated with metabolic and anabolic signaling in muscle, leading to a change in phenotype. The extent of these changes depends on the intensity, the duration, and the frequency of the exercise. The effect of exercise is also partly due to a decrease in inflammation, which has been shown to be closely related to the development of various diseases. Furthermore, it has been suggested that several phytochemicals contained in natural foods can improve nutrient metabolism and prevent protein degradation in the muscle.

## 1. Introduction

The incidence of metabolic syndrome is increasing worldwide. Metabolic syndrome refers to a collection of issues including visceral obesity, elevated blood glucose levels, dyslipidemia (elevated fasting triglycerides and low high-density lipoprotein (HDL) cholesterol levels), and hypertension. It leads to an increase in the risk of developing of cardiovascular disease (CVD), type 2 diabetes, and cancer. It can therefore occur as a predisease state. Thus, effective strategies preventing metabolic syndrome are required to decrease the incidence of diseases and promote healthy aging. 

**Figure 1:**
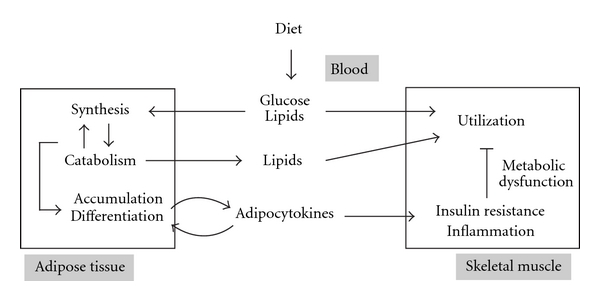
Crosstalk between skeletal muscle and adipose tissue in nutrient metabolism.

The development of metabolic syndrome is primary caused by a sedentary lifestyle and overnutrition; however, genetic characteristics are also involved to some extent. Daily physical activity directly influences obesity and metabolic syndrome associated with the metabolic function of skeletal muscle (Figure 1). Thus, dietary exercise, along with adequate diet, is well known to be one of the major preventive therapies against metabolic syndrome. In the last few decades, it has been shown, in epidemiological and experimental studies, that exercise reduces obesity, improves glucose tolerance, and decreases the risk of diabetes and CVD. The effects of exercise are brought about by elevated energy consumption, improvement of insulin sensitivity, and a reduction in inflammation. The molecular mechanisms underlying these benefits have been established. A single bout of exercise drastically changes various physiological parameters such as hormone production, blood flow, and the activity of the nervous system, in addition to altering the expression/activity of certain genes and proteins in the skeletal muscle. Further, regular exercise leads to permanent beneficial adaptations. This paper reviews evidence regarding the influence of exercise on the progress of metabolic syndrome along with its underlying molecular mechanisms. It particularly focuses on the skeletal muscle, a major metabolic organ, and describes the benefits of functional food factors combined with exercise therapy. 

## 2. Progression of Metabolic Syndrome due to a Sedentary Lifestyle

 Sedentary behavior and persistent low levels of physical activity are known to induce progression toward metabolic syndrome, type 2 diabetes, and CVD [1–5]. Energy consumption depends on the intensity and amount of physical activity; therefore, a sedentary life tends to result in a positive energy balance and leads to an accumulation of body fat. Adipose tissue secretes bioactive factors, adipocytokines, such as tumor necrosis factor alpha (TNF-*α*), plasminogen activator inhibitor, and resistin, into circulation. It has been considered that there is aclose relationship between these adipocytokines and health problems, such as obesity and metabolic and cardiovascular disorders, as they cause insulin resistance, injury to the endothelium, and inflammation. In addition, a sedentary lifestyle causes a decrease in resting energy metabolic capacity. This decrease may be due to atrophy of skeletal muscle, a major energy-consuming tissue in the body [6]. It has been reported that the loss of fat-free mass with inactivity and age explains a reduction in the resting metabolic rate (RMR) [7]. Muscle atrophy may be due to both muscle fiber atrophy and loss of complete muscle fibers [8, 9] due to several factors including the apoptosis of muscle cells [10], decreased differentiation of satellite cells [11], and reduced protein levels as a result of decreased protein synthesis and increased protein degradation [12]. The activity of enzymes involved in aerobic metabolism and glucose uptake in muscle is also decreased by inactivity and aging. Laboratory studies have shown that significant protein degradation is seen within 2 days of muscle immobilization leading to loss of muscle mass within 1 week [13]. Insulin-induced glucose uptake into the muscle is also reduced along with a reduction in its signaling pathway, within 2 days of immobilization [14]. At the same time, the activity of lipoprotein lipase (LPL), a protein important for controlling plasma triglyceride catabolism HDL cholesterol and other metabolic risk factors was lost [15]. Consistent with the decrease in LPL function, the clearance of plasma triglyceride by skeletal muscle was significantly decreased and plasma HDL cholesterol concentration declined [15]. 

Recently, it has been established that low-grade continuous inflammation and oxidative stress are associated with metabolic disorders and CVD [16–18]. Low levels of physical inactivity lead to chronic inflammation and oxidative stress in the skeletal muscle, the circulatory system, and other tissues. Some adipocytokines, such as TNF-*α* and interleukin-6 (IL-6), which are secreted from accumulated visceral adipose tissue can cause this inflammation. These proinflammatory cytokines impair glucose transport via the inhibition of insulin signal transduction. Insulin-induced activation of the insulin receptor (IR), phosphatidylinositol 3-kinase (PI3K), and Akt is prevented along with I*κ*B kinase (IKK) activation and degradation of I*κ*B in the muscle tissue [19–21]. Furthermore, IKK-*β* silencing prevents TNF-*α*-induced impairments in insulin action on Akt phosphorylation and glucose uptake [22]. Growing evidence suggests that additional adipocytokines including resistin, fatty-acid-binding protein (FABP), and visfatin have also induced insulin resistance associated with inflammation [23–25]. In addition, a reduction of circulating adiponectin, an adipocytokine with anti-inflammatory properties, occurs with obesity and leads to insulin resistance in skeletal muscle and liver [26–28]. A recent study clearly showed that adiponectin directly improves glucose and lipid metabolism along with mitochondria biogenesis and activation of the key metabolic modulators via adiponectin receptor 1 (AdipoR-1) in skeletal muscle [29]. This inflammation of skeletal muscle is caused by muscle inactivity even when body fat is low. Indeed, expression of TNF-*α* in skeletal muscle is elevated with insulin resistance in human [30]. This indicates that TNF-*α* generated from not only other cells but also myocytes disturbs insulin signaling. An increased level of oxidation of lipids, DNA, and proteins is also observed in muscles of sedentary subjects compared to that of active subjects [31–34]. Furthermore, continuous activation of intracellular oxidative-stress-sensitive factors such as the nuclear factor-kappa B (NF-*κ*B) and mitogen-activated protein kinase is seen in the muscle of sedentary men [35, 36]. Oxidative-stress is also strongly associated with development of insulin resistance in the skeletal muscle. Thus, oxidative-stress-induced insulin resistance in muscle leads to the initiation of diabetes and potentially late diabetic complications. It is without doubt that insulin sensitivity is inversely correlated with the plasma levels of free radicals in diabetic patients [17, 18]. Several studies have demonstrated that reactive oxygen species (ROS) impair insulin-mediated glucose uptake and storage by disrupting signaling control points such as glycogen synthase kinase-3, Akt phosphorylation, and actin remodeling [37–39]. In addition, we have recently found that 3-nitrotyrosine modification of adenylate kinase 1 (AK1), a key enzyme in synthesis; equilibration; regulation of adenine nucleotides is elevated in older muscle and that the modification of AK1 is involved in the impairment of glucose uptake via inhibition of AMP-activated protein kinase (AMPK). Furthermore, it has been suggested that metabolic regulation of adiponectin is associated with reduction of oxidative-stress in skeletal muscle [29]. 

 Inflammatory cytokines and ROS are also associated with protein degradation via activation of the ubiquitin-proteasome pathway. This is one of the major causes of protein degradation. *In vitro* studies have revealed that the addition of oxidants and TNF-*α* to myotubes increases protein degradation rates, ubiquitination of proteins such as myosin, and expression of the main components of the ubiquitin-proteasome pathway [40–42]. Muscle ring finger 1 (MuRF1) and atrogin-1 have been identified as the ubiquitin ligases whose activities increase during atrophy [43, 44]. NF-*κ*B can regulate the ubiquitin-proteasome proteolytic pathway through the induction of MuRF1 and proteasome expression [45–47]. Furthermore, it has been shown that the 20S proteasome can selectively degrade oxidatively modified proteins without ubiquitination [48, 49]. These observations suggest that protein degradation could be the link between oxidative stress, inflammatory cascade, and muscle atrophy. In fact, hyperactivity of NF-*κ*B and the ubiquitin-proteasome pathway has been identified as a major cause of aged-related muscle atrophy [50, 51]. 

## 3. Evidence for the Beneficial Effects of Exercise

Many large cohort studies have found that higher level of physical activity is associated with reduced risk of developing diabetes and CVD [52–58]. One of the first major trials to examine the effect of physical activity was the University of Pennsylvania Alumni Health Study [53]. In this study, the level of physical activity was found to be inversely related to the development of type 2 diabetes in 202 male subjects. The incidence declined by 6% for each 500 kcal increment in energy expenditure from less than 500 to 3500 kcal. In addition, the Osaka Health Survey [59] showed, in 444 men, that regular exercise, at least once a week, reduced the relative risk of type 2 diabetes to 0.75 compared with in those engaging in exercise less often. Subjects who engaged in intense exercise at least once a week, at weekends, exhibited further reduction of the multiple-adjusted relative risk of type 2 diabetes to 0.55 compared with sedentary subjects. Cardiorespiratory fitness is also protective against diabetes and metabolic syndrome [60, 61]. Several controlled trials support this effect of physical activity. Studies lasting for 3–12 months involving exercise sessions of 30–60 min in a week have shown that regular exercise reduces fat mass and improves insulin sensitivity without dietary caloric restriction in overweight men and women [62–66]. Moreover, regular exercise decreases plasma levels of triglyceride and HDL cholesterol and lowers blood pressure [67–69]. Consistent with the improvement, regular exercise reduces circulating adipocytokines, such as resistin, visfatin, and FABP, involving development in metabolic disorder and inflammation [70–72]. On the other hand, the effect of exercise on circulating adiponectin is not completely known. Several studies have suggested that the improvement in insulin sensitivity induced by regular exercise is not mediated by changes in plasma adiponectin [73–75]. However, the ratio of high-molecular-weight form to total adiponectin was increased by regular exercise and there was a positive correlation between the increase of the adiponectin ratio and the improvement of insulin sensitivity in older insulin-resistant adults [76]. In addition, it has been shown that muscle AdipoR-1 is elevated in response to physical exercise [77], which elevates metabolic signal transduction of adiponectin and then improves oxidative metabolism. Therefore, the regulation of these adipocytokines including adiponectin likely contributes to the prevention of metabolic syndrome by daily exercise. 

 Aerobic training has traditionally been adopted as the main form of exercise therapy in epidemiological and laboratory studies. However, recently, the inclusion of resistance training as an integral part of an exercise therapy program has recently been endorsed by the American Heart Association [78], the American College of Sports Medicine [79], and the American Diabetes Association [80]. Cross-sectional studies have shown that muscle mass is inversely associated with mortality [81] and the prevalence of metabolic syndrome [82], independent of cardiorespiratory fitness levels. Even in the elderly, resistance training increases muscle mass from, 7.4% to 10.0%, along with muscle strength after 10–16 weeks [83, 84]. One study demonstrated that twice-weekly resistance training could prevent age-associated loss of lean body mass (LBM) and RMR, which is closely correlated to losses in LBM [85]. Resistance training contributes to an elevation in RMR as a result of a greater muscle protein anabolism [86]. Theoretically, a gain of 1 kg in muscle mass should result in an increase of approximately 21 kcal in RMR. Thus, resistance training, when sustained over years or decades, translates into clinically important differences in daily energy expenditure and can prevent age-associated fat gains. However, resistance training can rather inhibit LBM loss when combined with dietary restriction in antimetabolic syndrome therapy. In several randomized control trials, where obese men were randomly assigned to either a diet-only group or a diet with resistance training group, LBM was preserved by exercise training [87–95]. Furthermore, there is a strong support for the notion that resistance training is at least as effective as aerobic training in reducing some major CVD risk factors. Findings from several studies demonstrate that resistance training significantly decreases glycosylated hemoglobin levels in people with an abnormal glucose metabolism and has a tendency to improve lipoprotein-lipid profiles [90, 96, 97], independent of changes in body weight or composition. 

## 4. Molecular Mechanisms Underlying the Benefits of Exercise

**Figure 2:**
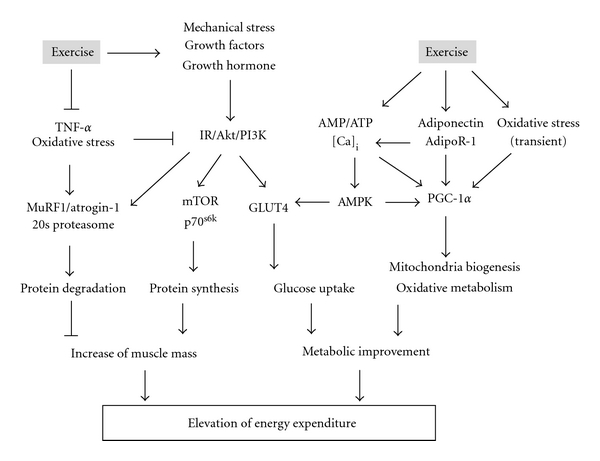
Molecular mechanism of muscle metabolic improvement due to exercise.

Exercise is accompanied by muscle contraction and subjects muscle cells to mechanical stress. This induces various intracellular signals. In addition, several factors such as hormones, growth factors, oxidative stress, and heat stress also affect signaling. Numerous human and animal studies have shown that metabolic improvement due to exercise occurs along with changes in the expression/activity of muscle proteins and alterations in their mRNA transcription (Figure 2). A single exercise bout improves glucose uptake in skeletal muscles via insulin-dependent and insulin-independent signal transduction mechanisms [98–101]. This effect is observed for several hours after exercise, often persisting until the next day. The increase in glucose uptake is caused by the translocation of the glucose transporter 4 (GLUT4) to the plasma membrane after activation of the IR/PI3K/Akt signaling pathway [102, 103]. Elevated AMPK activity and intracellular calcium levels can also induce GLUT4 translocation independent of the insulin signaling pathway [104, 105]. In addition, a considerable amount of attention has been given to the peroxisome proliferator-activated receptor gamma coactivator-1 alpha (PGC-1*α*) as a target for the prevention or treatment of metabolic syndrome. PGC-1*α* has been shown to have the central role in a family of transcriptional coactivators involved in aerobic metabolism and is activated by exercise [106]. Activation of PGC-1*α* alters the metabolic phenotype through interaction with nuclear respiratory factor and the peroxisome proliferator-activated receptor *α* [107, 108]. Improved understanding of the activation of the PGC-1*α* protein by exercise has implications beyond improving athletic performance [109, 110]. It may be target for the treatment of various diseases such as the mitochondrial myopathies and diabetes [111–113]. The activity/expression of LPL, a protein important for controlling triglyceride catabolism and cholesterol levels in plasma, is also elevated for 3 to 22 hours after exercise [114, 115]. These beneficial adaptations persist in humans and animals who perform regular exercise, independent of the acute effects of exercise. 

The molecular mechanisms underlying the maintenance of muscle mass have also been established. Exercise, particularly resistance exercise, promotes protein synthesis. Initiation of protein synthesis appears to be regulated by the Akt/the mammalian target of rapamycin (mTOR) signaling proteins. Akt phosphorylation regulates the catabolic pathway by preventing the induction of muscle-specific ubiquitin ligases such as atrogin-1 and MuRF1 [116] and activates the anabolic pathway by phosphorylating mTOR [117]. mTOR then initiates translation via the activation of translation regulators p70^s6k^ and the eukaryotic initiation factor-4E (eIF-4E) complex, following phosphorylation of eIF-4E-binding protein-1, one of the main translational inhibitors [118]. 

In addition, regular exercise can inhibit the apoptotic signaling pathway; this is associated with reducing oxidative stress and inflammation, and it results in the preservation of skeletal muscle fibers. This inhibitory effect results from the upregulation of antiapoptotic mediators, such as B-cell lymphoma/leukemia, X-linked inhibitors of apoptosis, and heat shock protein 70 and from the downregulation of proapoptotic mediators such as caspase-3 and Bax [119, 120]. A decrease in TNF-*α*, a factor that accelerates the caspase cascade, may be involved in antiapoptotic signaling, as regular exercise blunts TNF-*α* expression in aged muscle [121, 122]. Furthermore, the inhibition of TNF-*α* and oxidative stress would lead to a reduction in age-related muscle dysfunction, including prevention of protein degradation and impaired glucose uptake. 

The study of the mechanisms underlying the effects of exercise has been enhanced by the analysis of the function of microRNAs (miRNAs) in recent years. The miRNAs are small noncoding sections of RNA that regulate gene expression by degrading mRNA molecules or, more frequently in mammalian cells, inhibiting their translation [123, 124]. It has been suggested that miRNA-mediated gene regulation is a part of the fundamental mechanism of posttranscriptional regulation and may have diverse functional effects. In fact, 30% of protein-coding genes may be regulated by miRNAs [125]. Several of these miRNAs have been suggested to have a role in a wide range of biological processes, including development, cell death, carcinogenesis, and response to stress [126–129]. Some miRNAs, including miR-1, miR-133, and miR-206, which are referred to as myomiRs, have also been suggested to act as modulators of skeletal muscle function [130, 131]. We along with other researchers have shown that physical activity elevates muscle metabolism associated with PGC-1*α*, via regulation of some miRNAs [132, 133]. 

Furthermore, there is growing evidence that secreted proteins derived from muscle, also known as myokines, are elevated in plasma in response to exercise and regulate various functions of other organs. This regulation can mediate the benefits of exercise. Muscle-derived IL-6 is well known as a representative myokine that is markedly elevated in muscle and secreted into plasma following muscle contraction [134, 135]. This myokine could mediate some of the exercise-induced metabolic changes and anti-inflammatory effects in other organs such as the liver, adipose tissue, and blood vessels. Subsequently, other muscle-derived proteins, IL-15, and fibroblast growth factor-21 have been reported to regulate nutrient metabolism in other organs [135]. Furthermore, it has been shown that brain-derived neurotrophic factor is produced in skeletal muscle in response to contraction and has been suggested to increase fat oxidation in skeletal muscle in both an autocrine and paracrine fashion [135]. 

## 5. Effects of Combining Food Factors with Exercise Therapy

The potential effects of several food factors on muscle lipid metabolism in exercise have been investigated. Some of them have been found to accelerate lipid utilization; however, their efficacy is still controversial. A rate-limiting step in lipid metabolism in myocytes is the entry of long-chain fatty acids into the mitochondria. Carnitine palmitoyltransferase I (CPT I), located on the outer mitochondrial membrane, plays an important role in the entry of fatty acids into mitochondria. We found that a novel antioxidant astaxanthin limits the oxidative modification of CPT I by hexanoyl lysine [136]. This causes, along with elevated CPT I activity during exercise, acceleration of the reduction in body fat due to exercise training [136]. Ikeuchi et al. also showed that astaxanthin supplementation accelerates a catabolism in body fat along with a reduction of blood lactate [137]. Other food compounds with antioxidative capacities have also been identified. Catechin, one of the polyphenols contained in Japanese green tea, accelerates the utilization of fatty acids as an energy source for skeletal muscle contraction during exercise [138]. It has been suggested that the effect of catechin is related to the enhancement of *β*-oxidation activity and the level of fatty acid translocase/CD36 mRNA in muscle. An antioxidant, *α*-lipoic acid, increases glucose transport in the skeletal muscle. Intake of *α*-lipoic acid, combined with endurance exercise training, further accelerates glucose uptake and activity of the insulin signaling pathway compared with training alone [139]. Another compound that can affect energy metabolism is caffeine. It inhibits phosphodiesterase by promoting catecholamine release and increasing hormone-sensitive lipase activity [140]. This leads to an increase in circulating free fatty acids and a further improvement in endurance. Capsaicin, obtained from hot red peppers, is likely to enhance fat metabolism by increasing lipolytic hormones and promoting fat oxidation in the skeletal muscle [141].

However, growing evidence indicates that a large dose of dietary antioxidants prevents the adaptation generally seen as a result of regular exercise. Vitamin C supplementation (1 g/day, for 6 weeks) decreases the improvement of VO_2_ max associated with training [142]. Antioxidant vitamins and N-acetylcysteine reduced mitochondria biogenesis associated with the expression of PGC-1*α*, a key modulator of aerobic metabolism in skeletal muscle cells [143, 144]. More recently, in a prospective randomized intervention study, a combination of vitamin C (1000 mg/day) and vitamin E (400 IU/day) has been shown to inhibit the improvement of insulin sensitivity and elevation of plasma adiponectin along with the cancellation of PGC-1*α* induction in response to exercise training in healthy men [145]. These observations suggest that the intake of antioxidants is not always beneficial in counteracting muscle dysfunction related with inactivity and that oxidative stress is involved in signal transduction of exercise adaptation. 

It has been shown that the protein requirement for subjects performing resistance training is higher than that for sedentary individuals [146]. The daily recommended protein intake is estimated to be 1.4–1.8 g/kg for those performing resistance exercise when the intake of calories and carbohydrates is adequate [147]. However, it is not only the amount of protein, but also the timing of intake that is important for the efficient building of muscle. Eating protein immediately after exercise is more effective, in terms of increasing protein synthesis, compared with several hours later. The cross-sectional area of the quadriceps muscle after a 12-week resistance training program is greater [148]. Additionally, protein synthesis in muscle can be promoted by intake of proteins combined with carbohydrates via the actions of insulin as this accelerates the increase in muscle mass and strength [149]. 

In addition, it has been reported that the intake of amino acids and peptides is beneficial. Free amino acids and small peptide molecules do not need to be digested. Hence, absorption can be expected to be rapid. Amino acids are not only utilized in the synthesis of muscle protein but can also exert a variety of physiological effects. Attention has been focused on the effects of branched-chain amino acids (BCAAs), including valine, leucine, and isoleucine, which are known to be found in relatively high concentrations in both muscle proteins and food proteins. BCAAs are metabolized in the muscles and utilized as energy substrates. Their oxidation is enhanced during exercise by activation of branched-chain-*α*-keto acid dehydrogenase [150]. Therefore, when BCAAs are not supplied in the diet, muscle protein is catabolized to obtain them. Furthermore, dietary BCAAs modulate muscle protein metabolism to promote the synthesis and inhibit the degradation of proteins [151]. This results in an anabolic effect on muscles. Glutamine has also been reported to promote muscle growth by inhibiting protein degradation [152]. It is the most abundant free amino acid in muscle tissue, and its intake leads to an increase in myocyte volume and results in the stimulation of muscle growth. Glutamine is also found at relatively high concentrations in many other human tissues and has an important homeostatic role. Therefore, during catabolic states such as exercise, glutamine is released from skeletal muscle into the plasma to be utilized for maintenance of the glutamine level in other tissues [153]. *β*-hydroxy-*β*-methylbutyrate (*β*HMB) is a metabolite of the branched-chain amino acid leucine. It increases muscle mass by inhibiting the degradation of protein via its influence on the metabolism of branched-chain amino acids. A meta-analysis supported the use of *β*HMB while performing resistance exercise to augment LBM and strength [154]. Several studies have demonstrated that an intake of *β*HMB for at least 4 weeks achieved a greater increase in LBM or muscle power output [155]. 

## 6. Conclusion

Previously, numerous studies on prevention and development of metabolic syndrome have focused on effects of adipose tissue. On the other hand, because skeletal muscle plays an important role as a metabolic organ in the development of metabolic syndrome, recently these relationships have been established, as described in this paper. Exercise is the best tool for improvement in the muscle function. Daily exercise habit and physical fitness level are associated with a reduction in risk of metabolic syndrome, independently of body fat level. Therefore, although adequate diet is important for prevention and treatment of metabolic syndrome, regular exercise it needed for the therapies.
